# Supplementary material for: Loss of state transitions in Bryopsidales macroalgae and kleptoplastic sea slugs (Gastropoda, Sacoglossa)
Source: Commun Biol. 2025 Jun 5;8:869. doi: 10.1038/s42003-025-08305-3 (PMC12141491; doi:10.1038/s42003-025-08305-3)
Supplement: Supplementary file 2 — Supplementary Information [file 42003_2025_8305_MOESM2_ESM.pdf]

Supplementary information for Havurinne et al. 2025

Number of supplementary figures: 9

Number of supplementary tables: 2

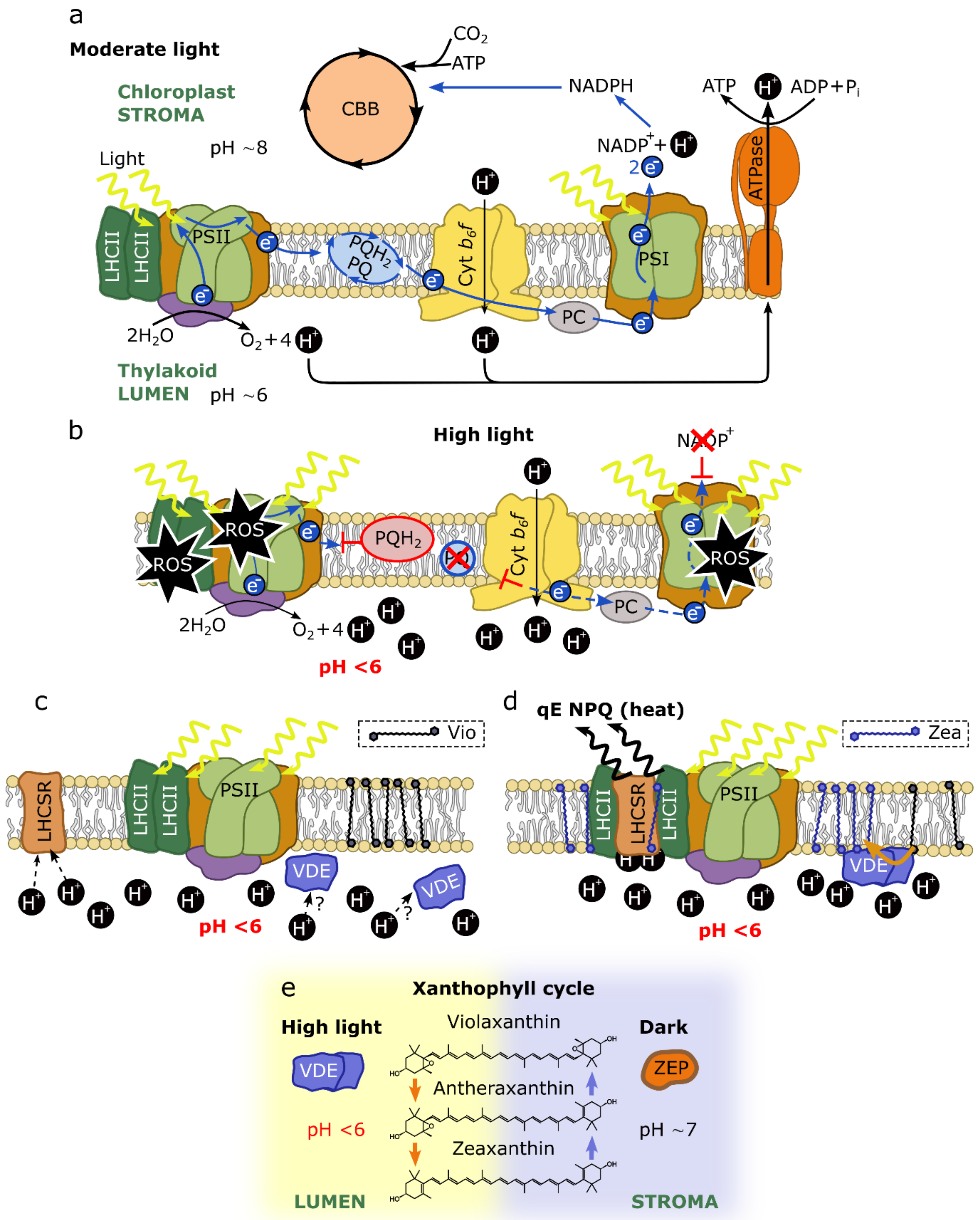

Supplementary Fig. 1. Induction of qE type NPQ and the conversion of violaxanthin to zeaxanthin in the xanthophyll cycle in green algae. (a) Photosynthetic electron transfer reactions in the thylakoid membrane of the chloroplasts during moderate intensity illumination. Light captured by the intrinsic antennae of PSII and PSI as well as their external light harvesting complexes, like light harvesting complex of PSII (LHCII), drive the photosynthetic electron transfer from water to NADPH. The release of protons by water splitting in PSII and during electron transfer reactions of the cytochrome b_6f complex (Cyt b_6f) mildly acidifies the thylakoid lumen and creates a proton motive force between the lumen and chloroplast stroma, which is

required for ATP production by the ATPase in photophosphorylation. ATP and NADPH are then used to fix CO_2 in the Calvin-Benson-Bassham cycle (CBB) to produce sugars. In moderate constant light there are very few limiting factors, allowing an uninterrupted electron flow from PSII to plastoquinone (PQ; reduced form plastoquinol PQH_2), through Cyt b_6f, plastocyanin (PC) and finally all the way through PSI. (b) High intensity illumination (high light) increases the electron donation by PSII, which can exceed the capacity of the rest of the electron transfer chain. The relatively slow electron transfer reactions in Cyt b_6f lead to a build-up of PQH_2, which blocks electron donation from PSII, increasing the production of harmful reactive oxygen species (ROS) in PSII and LHCII. The surge of electrons from PSI can also deplete the stromal electron acceptors like $NADP^+$, causing another hindrance which can lead to an increased production of ROS in PSI. However, the increased activities of PSII and Cyt b_6f immediately upon high light exposure further acidify the lumen pH, creating a salvatory link between high light exposure and photoprotection. (c-d) The stress-related light harvesting (LHCSR) proteins of green algae sense the drop in pH below 6 via protonation of specific residues on their luminal side, leading to their interaction with the LHCIIs. This enhances the dissipation of absorbed light energy as heat in the LHCIIs, which strongly competes with the photochemical electron transfer reactions of PSII and protects the electron transfer chain from receiving excessive light energy. This is the core process known as qE type non-photochemical quenching (NPQ) of excitation energy in green algae. qE can be further enhanced by another process involving the xanthophyll pigments violaxanthin (Vio) and zeaxanthin (Zea) residing in the thylakoid membrane. Free, inactive monomeric violaxanthin de-epoxidases (VDE) also sense the lumen pH <6, possibly via protonation, causing them to form active dimers that associate with the thylakoid membrane, where VDE can convert Vio to Zea in high light. Interaction between Zea and LHCSR/LHCII complexes increases the thermal dissipation of light energy, but is not strictly needed for qE induction in green algae. (e) The entire xanthophyll cycle also includes the intermediary xanthophyll antheraxanthin, which is converted to Zea in high light by VDE, and the cycle is reversed from Zea to Vio in the dark by the stromal zeaxanthin epoxidase (ZEP) in neutral to mildly basic pH.

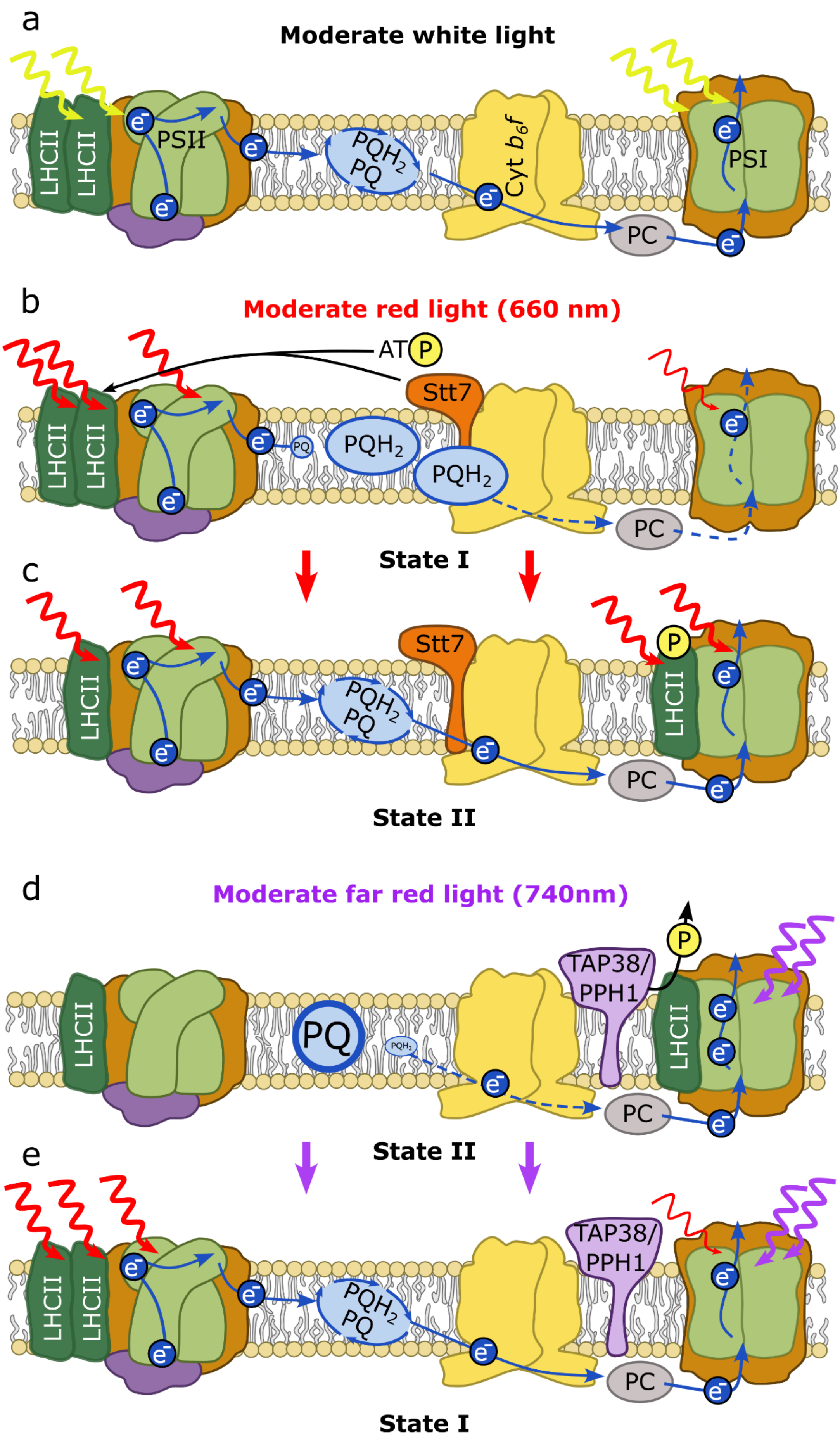
a
Moderate white light
LHCII
LHCII
PSII
PQH2
PQ
Cyt b6f
PSI
PC
b
Moderate red light (660 nm)
AT
P
Stt7
PQ
PQH2
PQH2
PC
State I
c
Stt7
P
LHCII
PQH2
PQ
PC
State II
d
Moderate far red light (740nm)
P
TAP38/
PPH1
LHCII
PQ
PQH2
PC
State II
e
TAP38/
PPH1
PQH2
PQ
PC
State I

Supplementary Fig. 2. State transitions in green algae. (a) The light harvesting between PSII and PSI in algae acclimated to specific polychromatic moderate intensity white light is well balanced; PSI can donate a steady stream of electrons originating from PSII via cytochrome b_6f complex (Cyt b_6f), and also the oxidized and reduced forms of plastoquinone (PQ, PQH_2, respectively) are in a balanced equilibrium. (b-c) When the algae are exposed to light that is predominantly absorbed by PSII and its light harvesting complexes (LHCIIs), like monochromatic red light of 660 nm, the light harvesting becomes unbalanced as there is comparatively less light energy to drive PSI electron transfer. PSI and Cyt b_6f can't keep up with the strong stream of electrons coming from PSII, leading to reduction of the PQ pool. The light acclimation state where all LHCIIs are attached to PSII, or State I, is not optimal for continuous photosynthesis in such light conditions. A state transition to a more suitable light harvesting arrangement is initiated by the increased occupancy of PQH_2 molecules in Cyt b_6f that activates the Stt7 kinase. Stt7 phosphorylates the mobile LHCII, which then moves and attaches to PSI, to light acclimation State 2. State 2 allows PSI to absorb more of the incident red light, restoring a well-balanced electron flow. (d-e) When algae in State 2 are exposed to light that is predominantly absorbed by the antennae of PSI, like far red light of 740 nm (that is exclusive to PSI), PSII does not donate enough electrons into the electron transfer chain to supply PSI. This causes a near complete oxidation of the PQ pool and the inactivation of the Stt7 kinase, allowing the TAP38/PPH1 phosphatase to dephosphorylate the mobile LHCII, leading to its detachment from PSI and return back to serve PSII and the reversal of the light acclimation state to State I again. LHCII can't absorb 740 nm far red light even in State I, but the light harvesting system is now more suited to balance out the electron transport between PSII and PSI if the algae are then exposed to e.g. red light on top of the far red light. It should be noted, that in nature illumination conditions leading to the extreme states 1 or 2 are not found, and therefore the system lingers in between.

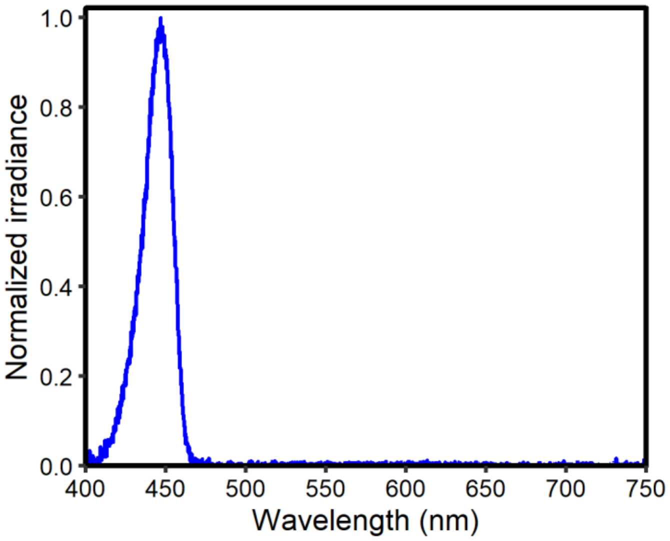

Supplementary Fig. 3. Spectrum of the excitation light used for the chlorophyll fluorescence measurements at 77K.

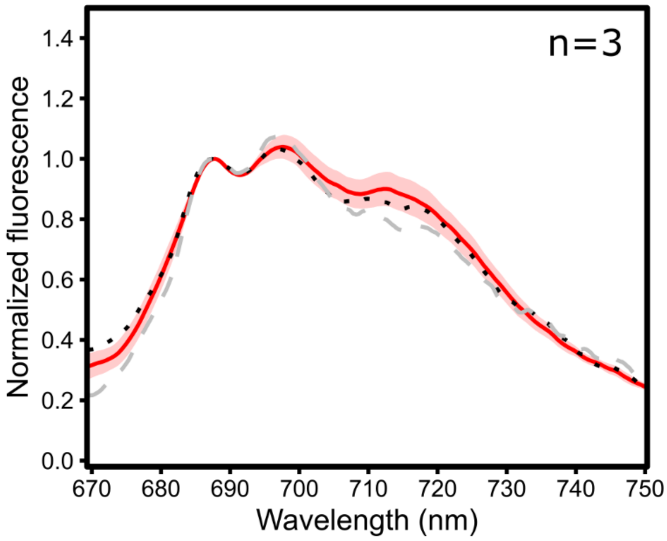

Supplementary Fig. 4. 77K fluorescence of *Elysia timida* (fed with *Acetabularia acetabulum*) samples exposed to high intensity PSII specific 660 nm red light (PPFD 100 µmol m^{-2} s^{-1}) for 15 min (red solid line). For the spectrum of the PSII light, see Fig. 2a. Excitation light was 450 nm. The line shows the mean from three biological replicates and the shaded area around the curve shows standard deviation. The data from the moderate intensity red (black dotted line) and far-red (dashed gray line) light treatments shown in Fig. 2l are replicated here for reference.

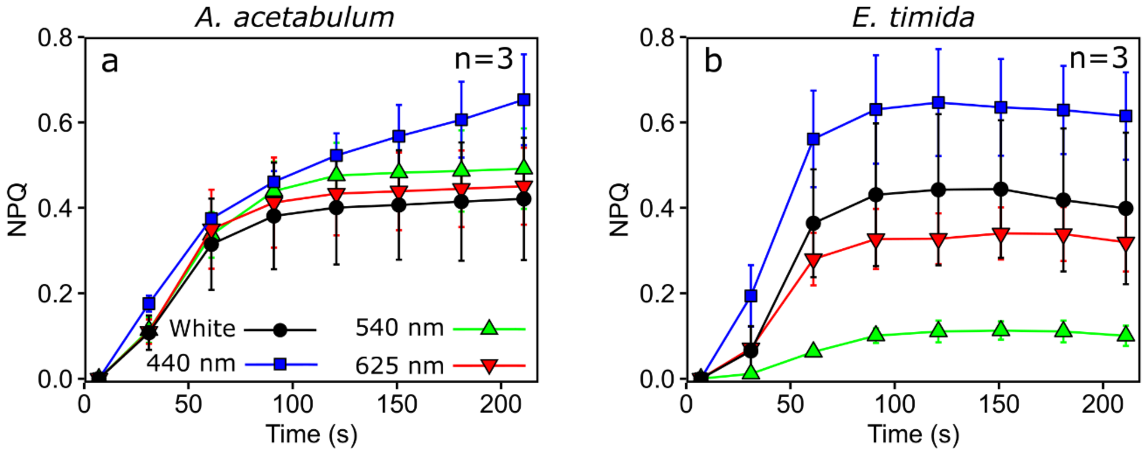

Supplementary Fig. 5. Non-photochemical quenching (NPQ) induction in (a) *Acetabularia acetabulum* and (b) *Elysia timida* measured with Multi-Color PAM fluorometer (Heinz Walz GmbH) in different colors of actinic light (PPFD 100 µmol photons m^{-2} s^{-1}; see legend for the wavelengths). The measuring light wavelength was the same as the actinic lights (625 nm in the white light treatment). The samples were dark acclimated for 20 min before measurements. Both the algae and the sea slugs were fixed in a drop of 1% alginate for the measurements, polymerized with 50 mM $CaCl_2$, which is a different method of fixation to that used in the experiments of the main text figures. The algae and sea slugs used in these measurements were of the same laboratory population as used in Havurinne and Tyystjärvi (2020)[30], and not the same population as the one used in the main manuscript text experiments. The differences in handling and fixation may themselves cause differences in NPQ between experiments in different studies. NPQ was calculated as F_M/F_M'-1. All data are means from three biological replicates and error bars show standard deviation.

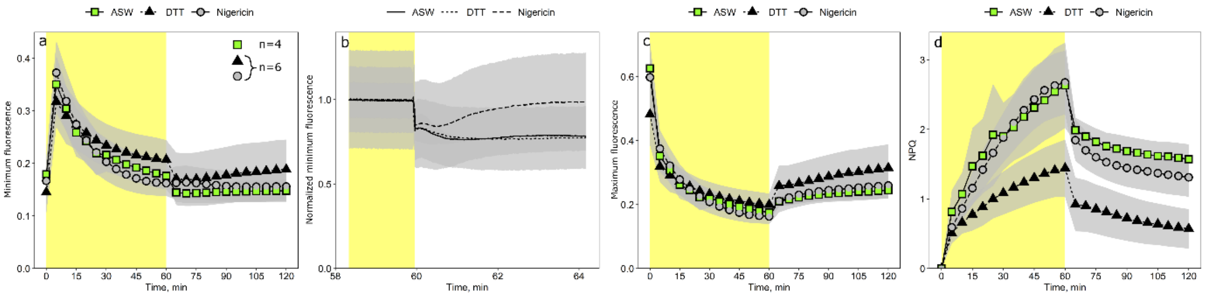

Supplementary Fig. 6. The effects of xanthophyll cycle and proton gradient inhibition by 10 mM DTT and 60 µM nigericin, respectively, on variable chlorophyll fluorescence and non-photochemical quenching (NPQ) in the true ulvophyte alga *Chaetomorpha* sp. in high light and subsequent recovery in the dark. Before the measurements, algae were kept in the dark for 30 min. (a-b) Base level (minimum) variable chlorophyll fluorescence kinetics; panel (b) details the base level fluorescence kinetics during the transition from light to dark; here fluorescence has been normalized to the level at the end of the light treatment. (c-d) Maximum fluorescence (F_M or F_M'), obtained by saturating light pulses, and NPQ (F_M/F_M'-1) during the treatment. The yellow background signifies the high light treatment (blue light; PPFD 500 µmol m^{-2} s^{-1}). The data are means from four (ASW) or six biological replicates (DTT, nigericin) and the gray shaded areas around the curves show standard deviation. ASW=artificial sea water (control conditions).

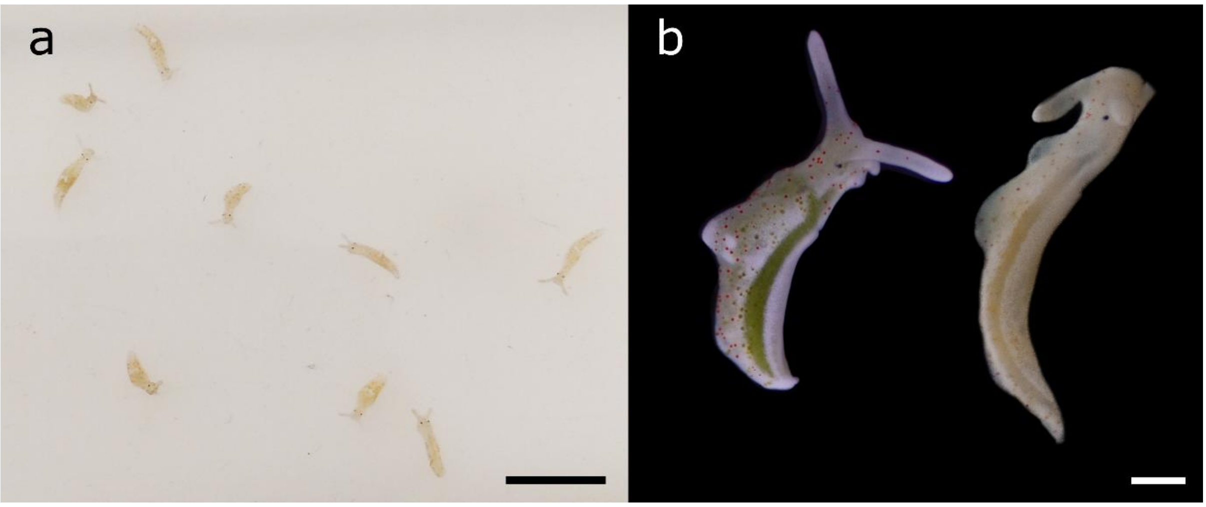

Supplementary Fig. 7. *Elysia timida* after bleaching in high light conditions in starvation. (a) Some of the individuals picked up for the re-feeding experiment after 11 days of bleaching in high light. Scale bar=10 mm. (b) A close-up of a green, freshly fed *E. timida* and a bleached individual. Scale bar=1mm.

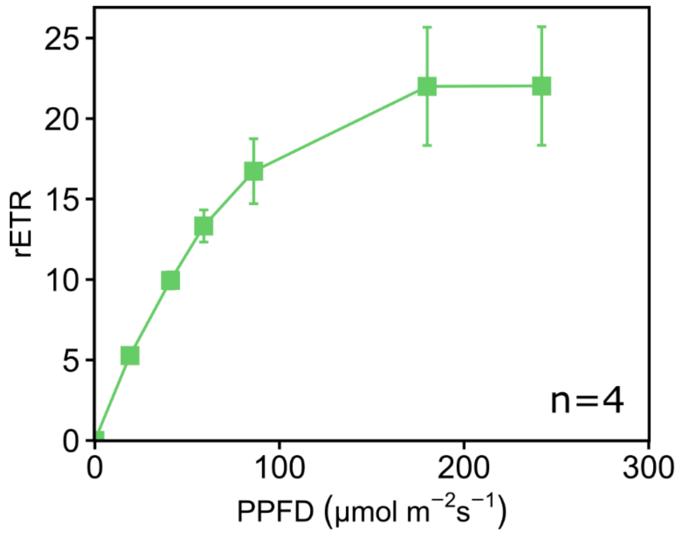

Supplementary Fig. 8. Photosynthetic electron transfer in *Elysia timida* fed with *Acetabularia acetabulum* at 35 PPT salinity (control growth conditions) determined by light response curves measured with a PAM fluorometer. The illumination at each PPFD lasted 60 s before determining the relative electron transfer rate (rETR) with a saturating light pulse. The data points are means from four biological replicates and the error bars show standard deviation.

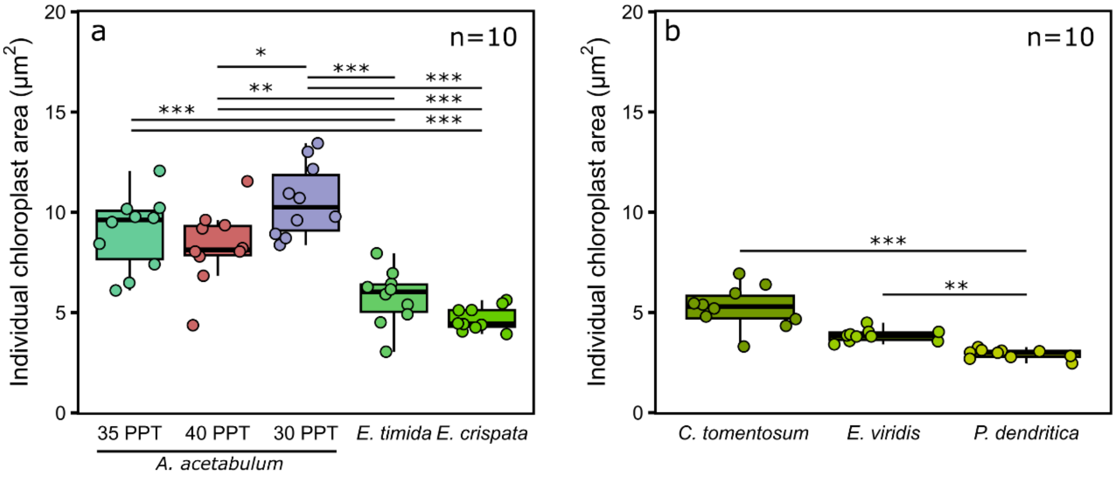

Supplementary Fig. 9. Estimations of the individual chloroplast areas of (a) *Acetabularia acetabulum* in different salinity treatments inside the alga or inside the sea slugs *Elysia timida* and *Elysia crispata*, and of (b) *Codium tomentosum* inside the alga and inside the sea slugs *Elysia viridis* and *Placida dendritica*, calculated based on confocal imaging of chlorophyll *a* auto-fluorescence. All *C. tomentosum* and sea slug samples were taken from 35 PPT salinity (growth conditions). Each individual datapoint shows the median chloroplast area from a single biological replicate (calculated from hundreds to thousands of identified chloroplasts), whereas the box plots show the medians and interquartile ranges from all 10 biological replicates. The whiskers indicate non-outlier maxima and minima. The asterisks mark significant differences between the indicated groups, determined by Kruskal-Wallis, followed by Dunn's test (* p-value <0.05, ** <0.01, *** <0.001).

Supplementary Table 1. The sea slug species used in the study and their respective growth conditions.

Species	Culture status	Origin	Growth medium	Sea salt type	Day/night cycle	Lamp	PPFD (µmol $m^{-2}s^{-1}$)	Temperature (°C)	Container system	Aeration/ water circulation	Feed stock alga
Elysia timida	Laboratory	Mediterranean (Elba, Italy)	ASW	Red Sea salt	12h/12h	NS12 35 W LED (Valoya, Helsinki, Finland)	40	19-22	3 L plastic boxes	Yes	*A. acetabulum*
Elysia crispata	Laboratory	Florida (USA)	ASW	Red Sea salt	12h/12h	V165 LED (Viparspectra, Richmond, CA, USA)	60-100	25	300 L life support system (LSS)	Yes	*A. acetabulum*, *B. plumosa*
Elysia viridis	Wild	Aguda beach, Vila Nova de Gaia, Portugal[a]; Labruge beach, Vila do Conde, Portugal[b]	ASW	Red Sea salt	12h/12h	V165 LED	60-100	18	300 L LSS	Yes	*C. tomentosum*
Placida dendritica	Wild	Labruge beach, Vila do Conde, Portugal[c]	ASW	Red Sea salt	12h/12h	V165 LED	60-100	18	300 L LSS	Yes	*C. tomentosum*

[a]Individuals used in Fig. 2; [b, c]Individuals used in Fig. 3, Fig. 7 and Supplementary Fig. 9; ASW, artificial sea water.

Supplementary Table 2. The algae used in the study and their respective growth conditions.

Species	Clade	Strain ID	Origin	Growth medium	Sea salt type	Day/night cycle	Light source	PPFD (µmol $m^{-2}s^{-1}$)	Temperature (°C)	Container system	Aeration/water circulation
Acetabularia acetabulum	Dasycladales	DI1	Mediterranean	ASW + f/2	Red Sea salt	12h/12h	NS12 35 W LED	40	19-22	1-10 L plastic boxes	No
Bryopsis plumosa	Bryopsidales	KU-0990 (KUMACC, Japan)	Japan	ASW + f/2	Red Sea salt	12h/12h	NS12 35 W LED	60	19-22	2 L glass bottles	Yes
Codium tomentosum	Bryopsidales	Wild	Aguda beach, Vila Nova de Gaia, Portugal[a]; Labruge beach, Vila do Conde, Portugal[b]	ASW	Red Sea salt	12h/12h	V165 LED	60-100	18	300 L life support system (LSS)	Yes
Caulerpa sp.	Bryopsidales	n.a.	Unknown[c]	Filtered natural sea water	Natural (Ria de Aveiro, Ilhavo, Portugal)	12h/12h	V165 LED	60-100	25	300 L LSS with other sea life	Yes
Chaetomorpha sp.	Cladophorales	n.a.	Unknown[c]	Filtered natural sea water	Natural	12h/12h	V165 LED	60-100	25	300 L LSS with other sea life	Yes
Cladophora sp.	Cladophorales	Wild	Tamargueira beach, Figueira da Foz, Portugal	ASW	Red Sea salt	12h/12h	NS12 35 W LED	100	18	0.5-1 L glass bottles	Yes
Ulva spp.	Ulvales	Wild	Ria de Aveiro, Ilhavo, Portugal	Filtered natural sea water	Natural	Natural January	Filtered sun light	250-500 on a clear day at 12:00 am	19	200 L plastic container	Yes

[a]Individuals used in Fig. 2; [b]Individuals used in Fig. 3, Fig. 7 and Supplementary Fig. 9; [c]spontaneous natural growth in laboratory coral reef aquariums; ASW, artificial sea water.
