# Supplementary material for: Loss of state transitions in Bryopsidales macroalgae and kleptoplastic sea slugs (Gastropoda, Sacoglossa)
Source: Commun Biol. 2025 Jun 5;8:869. doi: 10.1038/s42003-025-08305-3 (PMC12141491; doi:10.1038/s42003-025-08305-3)
Supplement: Supplementary file 3 — Reporting summary [file 42003_2025_8305_MOESM3_ESM.pdf]

nature portfolio

Corresponding author(s): Vesa Havurinne, Sónia Cruz

Last updated by author(s): May 12, 2025

Reporting Summary

Nature Portfolio wishes to improve the reproducibility of the work that we publish. This form provides structure for consistency and transparency in reporting. For further information on Nature Portfolio policies, see our Editorial Policies and the Editorial Policy Checklist.

Statistics

For all statistical analyses, confirm that the following items are present in the figure legend, table legend, main text, or Methods section.

n/a	Confirmed	
☐	☒	The exact sample size (*n*) for each experimental group/condition, given as a discrete number and unit of measurement
☐	☒	A statement on whether measurements were taken from distinct samples or whether the same sample was measured repeatedly
☐	☒	The statistical test(s) used AND whether they are one- or two-sided<br>*Only common tests should be described solely by name; describe more complex techniques in the Methods section.*
☒	☐	A description of all covariates tested
☐	☒	A description of any assumptions or corrections, such as tests of normality and adjustment for multiple comparisons
☐	☒	A full description of the statistical parameters including central tendency (e.g. means) or other basic estimates (e.g. regression coefficient) AND variation (e.g. standard deviation) or associated estimates of uncertainty (e.g. confidence intervals)
☐	☒	For null hypothesis testing, the test statistic (e.g. *F*, *t*, *r*) with confidence intervals, effect sizes, degrees of freedom and *P* value noted<br>*Give P values as exact values whenever suitable.*
☒	☐	For Bayesian analysis, information on the choice of priors and Markov chain Monte Carlo settings
☒	☐	For hierarchical and complex designs, identification of the appropriate level for tests and full reporting of outcomes
☒	☐	Estimates of effect sizes (e.g. Cohen's *d*, Pearson's *r*), indicating how they were calculated

Our web collection on statistics for biologists contains articles on many of the points above.

Software and code

Policy information about availability of computer code

Data collection	Light spectra and 77K fluorescence measurements data collection with OceanView 2.0 Software (Ocean Insight, Orlando, FL, USA); Imaging PAM fluorescence data collection with ImagingWin Software V2.56 (Heinz Walz GmbH, Effeltrich, Germany); Junior PAM fluorescence data collection with WinControl Software V3.32 (Heinz Walz GmbH, Effeltrich, Germany); Confocal microscope data collection with Leica Application Suite X LASX V.6.2.2.28360 (Leica Microsystems, Wetzlar, Germany).
Data analysis	MS Excel v.16.0 (MS Office 365; Microsoft Corporation, Redmond, WA, USA) was used to calculate means and standard deviations and to treat 77K fluorescence data. Fiji/ImageJ (ImageJ V. 1.54h+) was used to analyse microscope images with the StarDist plugin. Statistical analyses were done using R (v.4.2.3) in RStudio (2023.06.0+421), and figures were prepared with ggplot2 v3.4.1 package of R in conjunction with the open source software Inkscape.

For manuscripts utilizing custom algorithms or software that are central to the research but not yet described in published literature, software must be made available to editors and reviewers. We strongly encourage code deposition in a community repository (e.g. GitHub). See the Nature Portfolio guidelines for submitting code & software for further information.

Data

Policy information about availability of data

All manuscripts must include a data availability statement. This statement should provide the following information, where applicable:

- Accession codes, unique identifiers, or web links for publicly available datasets
- A description of any restrictions on data availability
- For clinical datasets or third party data, please ensure that the statement adheres to our policy

The source data for the main figures is available at https://doi.org/10.5281/zenodo.14179375. The original microscope images used in the study are available from the corresponding authors upon request.

Research involving human participants, their data, or biological material

Policy information about studies with human participants or human data. See also policy information about sex, gender (identity/presentation), and sexual orientation and race, ethnicity and racism.

Reporting on sex and gender	n/a
Reporting on race, ethnicity, or other socially relevant groupings	n/a
Population characteristics	n/a
Recruitment	n/a
Ethics oversight	n/a

Note that full information on the approval of the study protocol must also be provided in the manuscript.

Field-specific reporting

Please select the one below that is the best fit for your research. If you are not sure, read the appropriate sections before making your selection.

☒ Life sciences ☐ Behavioural & social sciences ☐ Ecological, evolutionary & environmental sciences

For a reference copy of the document with all sections, see nature.com/documents/nr-reporting-summary-flat.pdf

Life sciences study design

All studies must disclose on these points even when the disclosure is negative.

Sample size	A standard minimum of three biological replicates was used for all experiments. More biological replicates were used if the data was highly variable (with the consideration of biological material availability).
Data exclusions	No data were excluded.
Replication	In most cases the reproducibility was confirmed simply by using a minimum of three biological replicates for the experiments (see also "Sample size" above). In most cases the experiments required considerable time to perform, forcing us to do experiments with biological material separated by days or even months, and the results were still the same. All attempts at replication were successful.
Randomization	True randomization was not used in this study due to the nature of the biological material and its availability. Also the need to use similar sized sea slugs for the experiments imposed an unavoidable selection process. However, the samples for the experiments were taken from separate growth tanks/aquariums as often as possible. In the case of algae, the only selection criteria was that they looked healthy, but no special consideration was given to their age or other factors.
Blinding	True blinding was not realistically achievable in our study with the staff available in our lab. We note that this poses a weak point of our study, but stand firm behind our conclusions.

Reporting for specific materials, systems and methods

We require information from authors about some types of materials, experimental systems and methods used in many studies. Here, indicate whether each material, system or method listed is relevant to your study. If you are not sure if a list item applies to your research, read the appropriate section before selecting a response.

Materials & experimental systems

n/a	Involved in the study	
☒	☐	Antibodies
☒	☐	Eukaryotic cell lines
☒	☐	Palaeontology and archaeology
☐	☒	Animals and other organisms
☒	☐	Clinical data
☒	☐	Dual use research of concern
☐	☒	Plants

Methods

n/a	Involved in the study	
☒	☐	ChIP-seq
☒	☐	Flow cytometry
☒	☐	MRI-based neuroimaging

Animals and other research organisms

Policy information about studies involving animals; ARRIVE guidelines recommended for reporting animal research, and Sex and Gender in Research

Laboratory animals	Elysia timida (Mediterranean locality, Elba, Italy), Elysia crispata (locality Florida, USA). Ages of the animals varied. See Supplementary Table 1 of the manuscript for detailed information.
Wild animals	Elysia viridis and Placida dendritica were collected from the wild from different locations of the Portuguese coast and transported to our facilities within the same day and put to grow in life support systems running on artificial sea water. Individuals that were not used in the experiments were kept in the life support systems until they came to the end of their life cycle or they were released back to the sea. See Supplementary Table 1 of the manuscript for detailed information.
Reporting on sex	All animals used in the study are hermaphrodites.
Field-collected samples	E. viridis and P. dendritica were maintained in 300 L life support systems at 18 degrees celsius, 12/12h day/night cycle with a 60-100 umol (PPFD) light intensity.
Ethics oversight	This study was performed in accordance with EU legislation and directives concerning scientific research on animals, including the 3R principles. Ethical approval is not required for studies conducted with non-cephalopod invertebrates. Wild algae and sea slugs were collected from non-protected marine coastal areas. Laboratory-reared specimens used in this work were maintained in optimized life-support systems to ensure animal welfare.

Note that full information on the approval of the study protocol must also be provided in the manuscript.

Dual use research of concern

Policy information about dual use research of concern

Hazards

Could the accidental, deliberate or reckless misuse of agents or technologies generated in the work, or the application of information presented in the manuscript, pose a threat to:

No	Yes	
☒	☐	Public health
☒	☐	National security
☒	☐	Crops and/or livestock
☒	☐	Ecosystems
☒	☐	Any other significant area

Experiments of concern

Does the work involve any of these experiments of concern:

No	Yes	
☒	☐	Demonstrate how to render a vaccine ineffective
☒	☐	Confer resistance to therapeutically useful antibiotics or antiviral agents
☒	☐	Enhance the virulence of a pathogen or render a nonpathogen virulent
☒	☐	Increase transmissibility of a pathogen
☒	☐	Alter the host range of a pathogen
☒	☐	Enable evasion of diagnostic/detection modalities
☒	☐	Enable the weaponization of a biological agent or toxin
☒	☐	Any other potentially harmful combination of experiments and agents

Plants

Seed stocks	All relevant information about the algae strains used in the study are detailed in the Supplementary table 2 of the manuscript.
Novel plant genotypes	n/a
Authentication	The algae species/strains used in this study have been authenticated by either DNA-barcoding, morphological analysis, based on the culture collection data availability, or by a combination of multiple methods.
